# Multiparametric Cardiac Magnetic Resonance Assessment in Sickle Beta Thalassemia

**DOI:** 10.3390/diagnostics14070691

**Published:** 2024-03-26

**Authors:** Laura Pistoia, Antonella Meloni, Vincenzo Positano, Filomena Longo, Zelia Borsellino, Anna Spasiano, Riccardo Righi, Stefania Renne, Daniela Izzo, Ketty Savino, Sophie Mavrogeni, Emilio Quaia, Filippo Cademartiri, Alessia Pepe

**Affiliations:** 1 Unità Operativa Complessa Ricerca Clinica, Fondazione G. Monasterio CNR—Regione Toscana, 56124 Pisa, Italy; laura.pistoia@ftgm.it; 2 Department of Radiology, Fondazione G. Monasterio CNR—Regione Toscana, 56124 Pisa, Italy; antonella.meloni@ftgm.it (A.M.); positano@ftgm.it (V.P.); fcademartiri@ftgm.it (F.C.); 3 Bioengineering Unit, Fondazione G. Monasterio CNR—Regione Toscana, 56124 Pisa, Italy; 4 Unità Operativa Day Hospital della Talassemia e delle Emoglobinopatie, Azienda Ospedaliero-Universitaria “S. Anna”, 44124 Ferrara, Italy; filomena.longo@ospfe.it; 5 Unità Operativa Complessa Ematologia con Talassemia, ARNAS Civico “Benfratelli-Di Cristina”, 90134 Palermo, Italy; zelia.borsellino@arnascivico.it; 6 Unità Operativa Semplice Dipartimentale Malattie Rare del Globulo Rosso, Azienda Ospedaliera di Rilievo Nazionale “A. Cardarelli”, 80131 Napoli, Italy; spasiano.anna@tiscali.it; 7 Diagnostica per Immagini e Radiologia Interventistica, Ospedale del Delta, 44023 Ferrara, Italy; riccardo.righi@ausl.fe.it; 8 Struttura Complessa di Cardioradiologia-UTIC, Presidio Ospedaliero “Giovanni Paolo II”, 88046 Cosenza, Italy; stefania.renne@virgilio.it; 9 Unità Operativa Complessa di Cardiologia-UTIC, Presidio Ospedaliero “D.ssa Anastasia Guerriero”, 81025 Caserta, Italy; daniela.izzo1989@gmail.com; 10 Sezione di Cardiologia e Fisiopatologia Cardiovascolare, Dipartimento di Medicina e Chirurgia, Università degli Studi di Perugia, 06132 Perugia, Italy; ketty.savino@unipg.it; 11 Onassis Cardiac Surgery Center, 17674 Athens, Greece; sophie.mavrogeni@gmail.com; 12 Istituto di Radiologia, Dipartimento di Medicina, Università di Padova, 35128 Padova, Italy; emilio.quaia@aopd.veneto.it

**Keywords:** sickle beta-thalassemia, magnetic resonance imaging, iron overload

## Abstract

Cardiac involvement in sickle beta thalassemia (Sβ-thal) patients has been poorly investigated. We aimed to evaluate cardiac function and myocardial iron overload by cardiovascular magnetic resonance (CMR) in patients with Sβ-thal. One-hundred and eleven Sβ-thal patients consecutively enrolled in the Myocardial Iron Overload in Thalassemia (MIOT) network were studied and compared with 46 sickle cell anemia (SCA) patients and with 111 gender- and age- matched healthy volunteers. Cine images were acquired to quantify biventricular function. Myocardial iron overload (MIO) was assessed by the T2* technique, while macroscopic myocardial fibrosis was evaluated by the late gadolinium enhancement (LGE) technique. In Sβ-thal and SCA patients, the morphological and functional CMR parameters were not significantly different, except for the left atrial area and left ventricular (LV) stroke volume, indexed by body surface area (*p* = 0.023 and *p* = 0.048, respectively), which were significantly higher in SCA patients. No significant differences between the two groups were found in terms of myocardial iron overload and macroscopic myocardial fibrosis. When compared to healthy subjects, Sβ-thal patients showed significantly higher bi-atrial and biventricular parameters, except for LV ejection fraction, which was significantly lower. The CMR analysis confirmed that Sβ-thal and SCA patients are phenotypically similar. Since Sβ-thal patients showed markedly different morphological and functional indices from healthy subjects, it would be useful to identify Sβ-thal/SCA-specific bi-atrial and biventricular reference values.

## 1. Introduction

Sickle beta thalassemia (Sβ-thal) is characterized by the coinheritance of a sickle cell anemia gene and a beta thalassemia gene (β0 or β+) [1,2], presenting both a qualitative and quantitative biosynthetic anomaly of hemoglobin. This condition is relatively common in countries where beta thalassemia alleles are more prevalent, such as the Middle East, the Mediterranean region and the Indian subcontinent [3,4,5]. In Italy, where the beta thalassemia trait is frequent, Sβ-thal represents the most prevalent form of sickling syndromes. The clinical phenotype of this variant form of sickle cell disease (SCD) is characterized by a chronic hemolytic anemia with multiple cardiac and vascular complications [3,6,7]. The severity of symptoms and the treatment approach can vary, depending on the specific genetic mutations involved [8,9]. There is a lack of studies specifically documenting the phenotype of Sβ-thal patients; in fact, Sβ-thal patients have often been included in studies on homozygous SCD patients, based on a similar clinical course [10].

The organ-specific involvement in SCD and beta thalassemia patients has been extensively described. Cardiovascular impairment represents an important cause of morbidity and mortality both in SCD and in beta thalassemia [10,11]. Both syndromes lead to a chronic hemolysis-related anemia and a compensatory rise in blood volume, which enhances the cardiac output state [12]. This chronic increased workload can cause heart enlargement and potentially lead to high cardiac output cardiomyopathy [13,14]. The hemolytic anemia can be reduced with chronic red blood cell (RBC) transfusion therapy, which in turn can contribute to the development of a secondary state of iron overload, another potential factor stressing the cardiovascular system [15,16]. While beta thalassemia transfusion dependent (TD) patients are mainly characterized by an iron-related cardiomyopathy [17,18], cardiovascular involvement in SCD, in addition to hemolysis, typically includes vaso-occlusive crisis [19], as well as pulmonary hypertension [20,21] and a chronic inflammatory vasculopathy, leading to ischemia in various tissues and organs, including the heart [22,23,24,25].

Cardiac disease in SCD has been extensively described through a wide range of diagnostic modalities, such as Doppler echocardiography, scintigrafic techniques and, more recently, cardiac magnetic resonance (CMR) imaging [26,27,28]. Due to its multiparametric nature, CMR is a powerful method for in-depth evaluation and monitoring of myocardial structural and functional disorders in SCD patients [28,29]. CMR is the gold standard method in cardiology for quantifying biventricular volumes and function with excellent reproducibility [30]. Moreover, T2* CMR has emerged as a non-invasive and reproducible technique that is able to monitor cardiac involvement by iron overload as a possible complication of RBC transfusion therapy in SCD patients [31,32]. The T2* technique exploits paramagnetic iron compounds that cause local inhomogeneities of the magnetic field, shortening the T2* relaxation time proportionally to the iron concentration. Late gadolinium enhancement (LGE) CMR is a non-invasive valuable method for the identification of replacement myocardial fibrosis [33,34]. 

Cardiac involvement in Sβ-thal has been documented in only a few echocardiographic studies on Sβ-thal, in which patients were characterized by atrial and biventricular dilatation, right ventricular (RV) impairment [35], and systolic and diastolic left ventricular (LV) dysfunction [6,36]. Myocardial iron overload (MIO) is more frequent among beta thalassemia TD patients [37] than in patients with SCD [31,38]; however, with the growing life expectancy and extended use of chronic RBC transfusions, MIO is poised to become a more substantial clinical concern. To the best of our knowledge, there are no studies aiming to evaluate myocardial iron overload by CMR in patients with Sβ-thal, except for a report that has shown no evidence of cardiac iron in a small group (*N* = 10) of multitransfused Arab patients [39]. 

It is crucial for individuals with hemoglobinopathies to receive regular medical care, including cardiovascular assessment, in order to monitor and address any potential cardiac complications [10,40]. Early detection and intervention can help in improving the long-term outlook for these patients.

The primary purpose of this study was to systematically evaluate biventricular function and cardiac iron overload by CMR in a cohort of Sβ-thal patients, comparing them with a group of sickle cell anemia (SCA) patients. For a complete characterization of the heart of Sβ-thal patients, we also compared their functional and morphological MR parameters with those of a group of age- and sex-matched healthy subjects.

## 2. Materials and Methods

### 2.1. Study Population

The MIOT (Myocardial Iron Overload in Thalassemia) project was an Italian network constituted by 66 thalassemia centers and 10 magnetic resonance imaging (MRI) sites where CMR exams were performed using homogeneous, standardized and validated procedures [41]. The inclusion criteria of the MIOT project included (1) male and female patients, of all ages, with thalassemia syndromes or structural hemoglobin variants, requiring MRI to quantify cardiac and liver iron burden; (2) no absolute contraindications to MRI; (3) written informed consent; (4) written authorization for use and disclosure of protected health information. Clinical and instrumental data were collected in a web-based centralized database by all the MIOT centers [41]. The method of diagnosis of thalassemia syndromes or structural hemoglobin variants of the patients enrolled in the project was through hemoglobin analysis using high-performance liquid chromatography, electrophoresis, chromatography or molecular tests. The exclusion criteria of the MIOT project included (1) absolute contraindications to the CMR exam (non-MR conditional cardiac implantable electronic devices, catheters with metallic components, metallic foreign body in the eye, metallic fragments such as bullets, shotgun pellets, and metal shrapnel near great vessels or vital organs, cerebral artery aneurysm clips, magnetic dental implants, drug infusion devices); (2) patients with any other pathology or condition that, in the opinion of the researcher, excludes the patient from participation in the study. Patients with metal bones or joint implants that were not MRI compatible were also excluded.

We retrospectively compared the Sβ-thal patients and SCA patients consecutively enrolled in the project.

**Figure 1:**
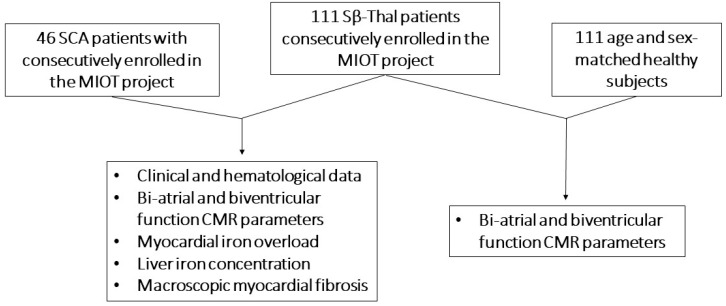
Study flow diagram. SCA = sickle cell anemia, Sβ-thal = sickle beta thalassemia, MIOT = myocardial iron overload in thalassemia, CMR = cardiac magnetic resonance.

Moreover, we considered a group of healthy volunteers, matched 1:1 with the Sβ-thal patients by gender and age, for the comparison of the bi-atrial and biventricular function MR parameters (Figure 1). Healthy subjects fulfilled the following criteria: (1) no history of cardiac or non-cardiac disease; (2) no cardiovascular risk factors; (3) normal electrocardiogram.

The study complied with the Declaration of Helsinki and was approved by the institutional ethics committee. Informed consent was obtained from all patients included in the study.

### 2.2. Magnetic Resonance Imaging (MRI)

MRI exams were performed on 1.5T scanners (GE Healthcare, Milwaukee, WI, USA; Siemens Healthineers, Erlangen, Germany;Philips, Best, The Netherlands). An eight-element cardiac phased-array receiver surface coil with breath-holding in end-expiration and ECG-gating were used for signal reception.

For the quantification of biventricular function parameters, steady-state free precession cines were acquired during 8 s breath holds in the vertical and horizontal long axis planes and in sequential 8 mm short-axis slices from the atrio-ventricular ring to the apex. Thirty cardiac phases were acquired per heart beat, and 10–14 slices were required to cover the ventricle over its entire extension. The most apical slice included was the first slice, which showed no blood pool at end-diastole. The most basal slice included was the one that showed a remaining part of the thick myocardium and was below the aortic valve. Images were analysed using MASS^®^ software Version 4.2 (Medis, Leiden, The Netherlands). Inter- and intra-observer reproducibility of biventricular function measurements have previously been shown to be high in healthy subjects [42] and in patients with hemoglobinopathies [43]. The analysis was based on the manual recognition of the endocardial and epicardial borders of the wall, at least in end-diastolic and end-systolic phases in each slice. Moreover, the papillary muscles and trabeculation within the LV and RV cavities were delineated and considered myocardial mass rather than part of the blood pool. For the calculation of biventricular end-diastolic and end-systolic volumes (EDV and ESV, respectively), no geometric assumption of the ventricle shape was needed. The stroke volume (SV) index was given by the difference between the EDV and ESV. The ejection fraction (EF) was calculated by the ratio between the SV and the EDV. Left and right atrial areas were measured from the four-chamber view projection in the ventricular end-systolic phase. The mass was calculated as the volume of the myocardium multiplied by its specific weight, which was assumed to be 1.05 g/cm^3^. The assessment of the RV mass was centralized in the coordinating center of the MIOT network and performed by a resident (D.I.) supervised by an expert operator in CMR (21 years of activity) (A.P.). Biventricular volumes and mass were indexed to the body surface area (BSA) and derived using the variation in the Dubois and Dubois formula [44]. 

The gradient-echo multi-echo T2* technique was used for iron overload assessment. For the heart, a multislice approach was used. Its reproducibility and its transferability within the MIOT network have been previously demonstrated [45], and the technique has been validated against autoptic hearts [46]. Three parallel short-axis views (basal, medium, and apical) of the left ventricle (LV) were acquired at nine echo times (Tes) [47]. For the liver, a single mid-transverse slice was obtained at nine Tes [48]. T2* images analysis was performed using a custom-written, previously validated software (HIPPOMIOT^®^ Version 1.0, Consiglio Nazionale delle Ricerche and Fondazione Toscana Gabriele Monasterio, Pisa, Italy) [49]. The software provided the T2* value for all the 16 segments of the LV according to the standard American Heart Association/American College of Cardiology model [50]. The global heart T2* value was obtained by averaging all segmental values. Hepatic T2* values were calculated in a circular region of interest [48] chosen in a homogeneous area of parenchyma without blood vessels, being converted into the liver iron concentration (LIC) using a Wood’s calibration curve [51].

LGE short-axis images were acquired 10–18 min after Gadobutrol (Gadovist^®^; Bayer; Berlin, Germany) intravenous administration at the standard dose of 0.2 mmoL/kg to detect the presence of macroscopic myocardial fibrosis. Additionally, vertical, horizontal, and oblique long-axis views were acquired. LGE was considered present when visualized in two different views [52]. LGE images were not acquired in patients with a glomerular filtration rate < 30 mL/min/1.73 m^2^ and in patients who refused.

### 2.3. Diagnostic Criteria

A T2* measurement of 20 ms was taken as a “conservative” normal value for segmental and global heart T2* values [18]. We identified four patterns of MIO: (1) no MIO (all segments with T2* ≥ 20 ms), (2) heterogeneous MIO (some segments with T2* ≥ 20 ms and some segments with T2* < 20 ms) and no significant global heart iron (global heart T2* ≥ 20 ms), (3) heterogeneous MIO with significant global heart iron (global heart T2* < 20 ms), (4) homogeneous MIO (all segments with T2* < 20 ms).

A MRI LIC ≥ 3 mg/g/dw was considered indicative of significant iron load [53].

### 2.4. Statistical Analysis

All data were analyzed using SPSS version 13.0. Continuous variables were described by mean ± standard deviation, while categorical variables were expressed as frequencies and percentages. The normality of distribution of the continuous parameters was assessed by using the Kolmogorov–Smirnov test.

Comparisons between groups were made by independent-sample *t*-tests for continuous variables with normal distribution, while, for continuous variables with non-normal distribution, a Mann–Whitney U test was applied. Χ^2^ testing was performed for non-continuous variables. Correlation analysis was performed by using Pearson’s or Spearman’s tests. 

In all tests, a 2-tailed probability value of 0.05 was considered statistically significant.

## 3. Results

### 3.1. Patients Characteristics

We retrospectively compared 111 patients with Sβ-thal (36.46 ± 14.63 years, 54 females) and 46 SCA patients (28.96 ± 14.32 years, 22 females) consecutively enrolled in the project. Notably, 48.6% of Sβ-thal patients and 65.2% of SCA patients were TD, receiving regular (≥4/year) simple (addition of new RBCs) RBC transfusions or regular exchange (replacement of the patient’s RBCs with new RBCs) RBC transfusions for at least one year. The term “non-transfusion dependent” (NTD) referred to patients who received no or sporadic blood/exchange transfusions. All Sβ-thal patients were Caucasian, while 64% of SCA patients were Caucasian, 31% were Black and 5% were mixed-race. 

### 3.2. Clinical and MRI Findings in Sβ-Thal Patients

The clinical and hematological data of Sβ-thal NTD and TD patients are summarized in Table 1. NTD Sβ-thal patients had significantly higher hemoglobin F (HbF) and hemoglobin S (HbS) levels than Sβ-thal TD patients, and they were more frequently treated with hydroxyurea (HU) therapy (*p* < 0.0001 in all cases). TD patients had a mean serum ferritin value that was significantly higher and resulted more frequently in chelation than NTD patients (*p* < 0.0001 in both cases). No differences were found in age, hemoglobin (Hb) levels, lactate dehydrogenase (LDH), serum creatinine, frequency of splenectomy, and history of pulmonary hypertension. 

Sβ-thal patients who performed HU therapy, compared to patients who did not, showed higher levels of HbF (15.76 ± 8.71% vs. 5.01 ± 4.28%, *p* < 0.0001) and HbS (62.43 ± 11.77% vs. 44.84 ± 20.03%, *p* < 0.0001). Moreover, compared to those who were non-chelated, chelated patients showed lower levels of HbS (48.77 ± 18.8% vs. 63.72 ± 11.68%, *p* < 0.0001) and a higher serum ferritin level (1571.79 ± 1610.90 ng/mL vs. 566.14 ± 462.52 ng/mL, *p* < 0.0001).

Table 2 shows the MRI findings in Sβ-thal NTD and TD patients. The two groups showed comparable values of biventricular function and bi-atrial MR parameters, except for RV mass index, which was significantly higher in NTD patients. 

Mean global heart T2* in Sβ-thal patients was 36.65 ± 6.68 ms, and 1.8% of patients (*N* = 2) had a global heart T2* < 20 ms. Thirty-six percent of Sβ-thal patients had at least 1 segment with T2* < 20 ms. No significant differences were found between TD and NTD patients in terms of global heart T2* values, and frequency of the different patterns of cardiac iron overload. A heterogeneous MIO with no significant global heart iron was found in 28.1% of NTD patients and in 40.7% of TD Sβ-thal patients.

The mean MRI LIC in Sβ-thal patients was 5.63 ± 6.02 mg/g/dw, and 53.2% of patients had a LIC > 3 mg/g/dw. LIC showed a direct and significative correlation with ferritin levels (R = 0.752, *p* < 0.0001). Compared to non-chelated patients, chelated patients showed higher levels of MRI LIC (6.8 ± 6.97 mg/g dw vs. 4.26 ± 4.55 mg/g dw, *p* = 0.045) and were more frequently transfused (72.9% vs. 22.9% *p* < 0.0001). TD Sβ-thal patients showed a higher MRI LIC and a higher frequency of patients with a LIC > 3 mg/g dw (*p* = 0.017 in both cases) than the NTD group. 

The percentage of Sβ-thal patients with replacement myocardial fibrosis by LGE was 17.9, and no significant difference was observed between TD and NTD Sβ-thal patients. 

Male Sβ-thal patients, compared to females, showed a significatively higher LV EDV index (95.0 ± 22.3 vs. 85.0 ± 15.5, *p* = 0.009), SV index (57.9 ± 11.8 vs. 50.6 ± 12.6, *p* = 0.003), cardiac index (4.2 ± 1.2 vs. 3.6 ± 1.1, *p* = 0.004), LV mass index (68.8 ± 12.8 vs. 53.8 ± 12.9, *p* < 0.0001), and RV EDV index (88.9 ± 20.6 vs. 78.0 ± 17.1, *p* = 0.004). No significant differences between sexes were found in other functional and morphological CMR parameters. A significantly lower global heart T2* was found in males (35.8 ± 4.8 vs. 37.6 ± 8.2, *p* = 0.016), and the LIC and the frequency of patients with replacement myocardial fibrosis by LGE were comparable in the two groups.

Age showed a significative correlation with cardiac index (R = −0.355, *p* < 0.0001) and with mean global heart T2* (R = 0.290, *p* = 0.002). No correlation was found between age and other morphological and functional CMR parameters, LIC and replacement myocardial fibrosis by LGE.

### 3.3. Comparison of Clinical and MRI Findings between Sβ-Thal and SCA Patients

Table 3 describes the clinical and hematological parameters of Sβ-thal and SCA patients. The two groups had comparable values of Hb, HbF, HbS, serum ferritin, LDH and serum creatinine. Sβ-thal patients were older than SCA patients (*p* = 0.004), and they were splenectomized more frequently (*p* < 0.0001) and treated with HU therapy (*p* = 0.005). There were no significant differences between Sβ-thal and SCA patients in the number of chelated patients, TD patients, or the number of patients with a history of pulmonary hypertension. 

Age resulted significantly higher in splenectomized patients (37.23 ± 12.76 vs. 30.66 ± 16.64, *p* = 0.018) and in patients treated with HU therapy (37.78 ± 10.82 vs. 29.03 ± 17.29, *p* = 0.001).

Table 4 shows MRI parameters in Sβ-thal and SCA patients. In the two groups, the biventricular EDV and ESV, EF and mass, as indexed by BSA, were not significantly different. Moreover, the cardiac index was not significantly different between the groups. SCA patients compared to Sβ-thal showed a significantly higher left atrial area index and SV index (*p* = 0.023 and *p* = 0.048, respectively). Since the left atrial area index and SV index were not associated with age, we did not apply a correction model.

Patients with a global heart T2* < 20 ms (*N* = 2; both patients were TD) were only observed among the Sβ-thal group. Respectively, Sβ-thal and SCA groups showed 34.2% and 21.7% of patients with a heterogeneous MIO and no significant global heart iron. No significant differences between the two groups were found in terms of global heart T2* < 20 ms, frequency of patients with at least one segment with T2* < 20 ms, and different patterns of myocardial iron overload and replacement myocardial fibrosis by LGE. The two groups showed comparable MRI LIC values and frequencies of patients with a MRI LIC > 3 mg/g dw.

### 3.4. Comparison of Bi-Atrial and Biventricular Function MR Parameters between Sβ-Thal Patients and Healthy Controls

**Figure 2:**
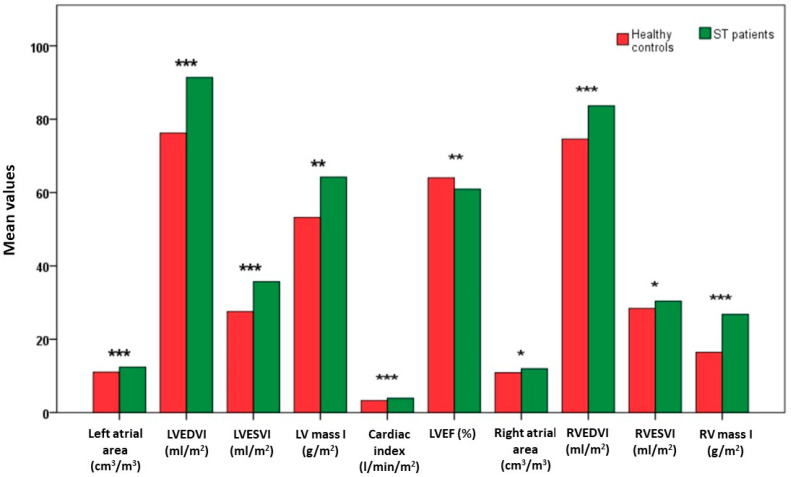
Comparison of bi-atrial and biventricular function MR parameters between Sβ-thal patients and healthy controls. Legend for the significant differences between the two groups: * *p* < 0.01, ** *p* = 0.001, *** *p* < 0.0001. ST = Sβ-thalassemia, LV = left ventricular, EDVI = end-diastolic volume index, ESVI = end-systolic volume index, EF = ejection fraction, RV = right ventricular.

Bi-atrial and biventricular function MR parameters of the 111 Sβ-thal patients were compared with those of 111 healthy volunteers, matched 1:1 by gender and age. Figure 2 represents graphically the morphological and functional MR parameters in Sβ-thal patients and healthy subjects. Left and right atrial areas were significantly larger in Sβ-thal patients than in healthy controls (*p* < 0.0001 and *p* < 0.01, respectively). All biventricular volume indexes were significantly larger in Sβ-thal patients than in healthy subjects (*p* < 0.0001 in all cases, except for *p* < 0.01 for RV end-systolic volume index). Moreover, cardiac index (*p* < 0.0001) and left (*p* = 0.001) and right (*p* < 0.0001) mass indexes were significantly higher in Sβ-thal patients than in healthy controls. The LVEF results were significantly lower in Sβ-thal patients than in the healthy group.

## 4. Discussion

TD and NTD Sβ-thal patients showed some clinical and hematological differences that were directly related to the RBC transfusion dependence and independence state. Moreover, NTD patients had a higher RV mass index, a sign of negative cardiac remodelling as a consequence of the non-transfusion dependence state, reflecting more sensitivity in the RV to the high cardiac output state. Compared to those non-chelated, chelated Sβ-thal patients showed lower HbS and greater LIC and ferritin levels, potentially because they were more frequently transfused. In line with previous studies on the general population [30] and patients with hemoglobinopathies [54], male Sβ-thal patients showed higher biventricular volumes than females, probably reflecting the intrinsic difference by gender in the cardiac chamber size. We also found that patients with Sβ-thal exhibit a reduction of cardiac index with age, indicating the natural and progressive decline in cardiac performance in the aging population, as reported in previous studies [55]. 

The morphological and functional CMR analysis in Sβ-thal patients in comparison with SCA patients showed a significantly lower left atrial area and SV index in Sβ-thal. A possible explanation of this difference could be the higher frequency of patients treated with HU among Sβ-thal patients. Accordingly, an Arab study showed that Sβ-thal were more likely to be on HU therapy than SCD homozygous patients, noting that they featured a more severe phenotype [3]. Moreover, previous studies showed a lower prevalence of atrial dilation in Sβ-thal than SCD homozygous patients [36,56]. It is known that the atrial myocardium is more sensitive to iron deposition and anemia than the ventricle [40], and this could explain the higher left atrial area in the less frequently HU-treated SCA group, as well as the absence of significantly different biventricular volumes between Sβ-thal and SCA patients.

To our knowledge, the only study with the aim to systematically evaluate MIO in Sβ-thal [39] included 10 multitransfused Arab patients, and none of them showed iron overload, as measured in the LV septum. In the present study, a segmental approach was used to assess MIO, with four different patterns of MIO being identified. Thanks to this more sensitive method, a heterogeneous MIO with no significant global heart iron was found in a considerable number of TD and NTD patients with Sβ-thal. Moreover, two patients showed a pathological mean global T2*, and one of them had a homogeneous MIO pattern. We also observed that T2* is directly related to age, but since the mean global cardiac T2* is within the normal range, this association is not clinically relevant. No significant differences were found in terms of MIO between Sβ-thal and SCA groups. Even in SCA patients, the segmental analysis identified a group with heterogeneous iron overload with global T2* > 20 ms, but a significant global iron burden (global heart < 20 ms) was not present in any of the patients we observed. It is known that cardiac iron overload is rarely found in SCD patients, being predominatly associated with poor control of total iron stores [31]. Despite the patient population of the MIOT project being well treated and constantly monitored, the more sensitive segmental approach was able to identify MIO at an early stage. Conversely, in about a half of SCA and Sβ-thal patients, we observed liver iron overload, which is consistent with previous studies [39,57], as it is known that liver iron overload occurs earlier in comparison with MIO. 

For a complete characterization of the heart of Sβ-thal patients, we also compared their bi-atrial and biventricular function CMR parameters with those of sex and age-matched healthy subjects. Accordingly, with previous echocardiographic studies [6,35], all morphological and functional indices were significantly increased in Sβ-thal patients rather than in healthy controls. Only the LVEF result was significantly lower in patients, as previously observed by Aessopos et al. [6], and, although the LVEF was within normal limits, it seems to be indicative of a subtle systolic LV dysfunction.

### Limitations

At the time of the current study, T1 and T2 mapping were not implemented in the MIOT network. In recent years, the native T1 mapping technique has been proposed as a complementary tool to the T2* technique thanks to its improved sensitivity in detecting changes associated with mild or early MIO [58]. In thalassemia patients, T2 mapping does not offer any advantage in terms of sensitivity for MIO assessment, but it does detect subclinical myocardial inflammation [59] and an increased myocardial extracellular volume, which potentially reflects diffuse interstitial fibrosis associated with MIO and heart failure [60]. The application of the above-mentioned technique on the Sβ-thal patients could open new horizons.

Unfortunately, NT-pro-BNP and troponin values, as indicators of changes in myocardial damage, were not systematically evaluated. The availability of the CMR by T2* and LGE techniques is restricted in the emergency setting, but the CMR is regularly performed for assessing myocardial iron and replacement fibrosis in order to optimize and tailor the management of the patients in countries at middle and high income. Due to cost and logistic reasons, the availability of the CMR technique is limited in low-income countries.

Another limitation of this study is that we did not have sufficient information on the quantitative β-globin defect of Sβ-thal patients (β0 or β+ mutation), and we also did not assess the hemoglobin A percentage, which could have been useful in better characterizing our population. Nevertheless, a recent study compared echocardiographic parameters of a group of Sβ-thal patients with β+ thalassemia mutation with those with β0 mutation [36]. They concluded that cardiac involvement in patients with sickle beta thalassemia does not appear to depend on the type of beta mutation, but the presence of signs of myocardial remodelling appears to be linked to multiorgan deficiency.

## 5. Conclusions

In conclusion, the clinical phenotype of Sβ-thal patients seemed to be only partially influenced by RBC transfusions, with NTD and TD groups showing some hematological differences and being comparable in terms of bi-atrial and biventricular MR parameters, except for RV mass index, which was higher in NTD patients. Despite the low frequency of patients with significant cardiac iron burden, the cardiac T2* segmental analysis identified a consistent group with heterogeneous iron overload. Moreover, the CMR analysis confirmed that the heart of Sβ-thal and SCA patients is phenotypically similar both in terms of biventricular function and cardiac iron.

As the lifespan of patients with hemoglobinopathies is increasing and cardiac iron and cardiovascular abnormalities have more time to develop, multiparametric cardiac characterization by CMR can provide important information for healthcare providers to optimize and tailor the management of these patients. A segmental CMR evaluation of MIO may help in identifying the early stages of this phenomenon in the Sβ-thal patient population. 

Since Sβ-thal patients showed markedly different morphological and functional indices from healthy volunteers, it would be useful to establish specific bi-atrial and biventricular reference values for Sβ-thal/SCA patients, as already performed for patients with thalassemia major [55], preventing the possibility of misdiagnosis of cardiomyopathy in patients with SCD.

## Figures and Tables

**Table 1:** Clinical and hematological data in sickle β-Thalassemia NTD and TD patients.

	NTD Sβ-Thal Patients *N* = 57	TD Sβ-Thal Patients *N* = 54	*p* Value
Sex, M/F	31/26	26/28	0.51
Age, years	37.3 ± 11.1	35.6 ± 17.7	0.62
Hemoglobin, g/dL	9.6 ± 1.6	9.3 ± 1.1	0.25
Hemoglobin F, %	15.3 ± 9.6	7.6 ± 6.2	<0.0001
Hemoglobin S, %	67.3 ± 9.1	43.3 ± 15.8	<0.0001
Serum ferritin, ng/mL	753.7 ± 893.0	1505.4 ± 1558.5	<0.0001
Lactate dehydrogenase, mg/dL	663.0 ± 237.6	650.7 ± 311.1	0.84
Serum creatinine, mg/dL	0.6 ± 0.2	0.7 ± 0.3	0.65
Splenectomy, *N* (%)	35 (61.4)	38 (70.4)	0.32
Chelation therapy, *N* (%)	16 (30.2)	43 (79.6)	<0.0001
Hydroxyurea therapy, *N* (%)	40 (85.1)	17 (36.2)	<0.0001
History of pulmonary hypertension, *N* (%)	1 (1.8)	0 (0.0)	0.32

NTD = non-transfusion dependent, TD = transfusion dependent, Sβ-thal = Sβ-thalassemia, *N* = number, M = male, F = female.

**Table 2:** Comparison of MRI findings between NTD and TD Sβ-Thal patients.

	NTD Sβ-Thal Patients	TD Sβ-Thal Patients	*p* Value
Left atrial area (cm^2^/m^2^)	12.3 ± 1.9	13.0 ± 3.1	0.65
Right atrial area (cm^2^/m^2^)	11.7 ± 2.0	12.0 ± 2.1	0.59
LV EDVI (mL/m^2^)	91.7 ± 22.3	88.2 ± 16.6	0.38
LV ESVI (mL/m^2^)	36.7 ± 15.9	35.1 ± 10.5	0.56
LV mass index (g/m^2^)	63.2 ± 14.8	59.3 ± 14.8	0.18
SVI (mL/m^2^)	55.4 ± 14.0	52.93 ± 11.0	0.31
Cardiac index (L/min/m^2^)	4.0 ± 1.1	4.0 ± 1.3	0.47
LV EF (%)	62.2 ± 6.4	59.8 ± 7.9	0.09
RV EDVI (mL/m^2^)	83.8 ± 22.9	83.2 ± 15.2	0.96
RV ESVI (mL/m^2^)	32.1 ± 15.2	31.8 ± 7.7	0.37
RV mass index (g/m^2^)	27.9 ± 8.8	21.6 ± 8.4	0.001
RV EF (%)	63.2 ± 9.3	60.8 ± 6.8	0.14
Global Heart T2* (ms)	37.3 ± 5.2	35.9 ± 7.9	0.59
Global Heart T2* < 20 ms. *N* (%)	0 (0.0)	2 (3.7)	0.14
At least 1 segment with T2* < 20 ms. *N* (%)	16 (28.1)	24 (44.4)	0.07
No MIO. *N* (%)	41 (71.9)	30 (55.6)	0.07
Heterogeneous MIO and no significant global heart iron. *N* (%)	16 (28.1)	22 (40.7)	0.16
Heterogeneous MIO and significant global heart iron. *N* (%)	0 (0.0)	1 (1.9)	0.30
Homogeneous MIO. *N* (%)	0 (0.0)	1 (1.9)	0.30
MRI LIC (mg/g dw)	3.9 ± 3.5	7.5 ± 7.5	0.017
MRI LIC > 3 mg/g dw. *N* (%)	24 (42.1)	35 (64.8)	0.017
Myocardial fibrosis by LGE. *N* (%) (*N* = 67)	7 (17.5)	5 (18.5)	0.92

NTD = non-transfusion dependent, TD = transfusion dependent, Sβ-thal = Sβ-thalassemia, *N* = number, LV = left ventricular, EDVI = end-diastolic volume index, ESVI = end-systolic volume index, SVI = stroke volume index, EF = ejection fraction, RV = right ventricular, MIO = myocardial iron overload, LIC = liver iron concentration, LGE = late gadolinium enhancement.

**Table 3:** Clinical and hematological data in sickle β-Thalassemia and SCA patients.

	Sβ-Thal Patients *N* = 111	SCA-Patients *N* = 46	*p* Value
Sex, M/F	57/54	24/22	0.93
Age, years	36.5 ± 14.6	29.0 ± 14.3	0.004
Hemoglobin, g/dL	9.4 ± 1.4	9.7 ± 1.3	0.49
Hemoglobin F, %	11.5 ± 9.0	10.3 ± 9.8	0.36
Hemoglobin S, %	55.4 ± 17.6	51.5 ± 17.6	0.33
Serum ferritin, ng/mL	1122.7 ± 1313.8	1353.3 ± 1501.1	0.41
Lactate dehydrogenase, mg/dL	657.0 ± 274.3	663.1 ± 269.0	0.99
Serum creatinine, mg/dL	0.7 ± 0.2	0.7 ± 0.2	0.68
Splenectomy, *N* (%)	73 (65.8)	13 (28.9)	<0.0001
Chelation therapy, *N* (%)	59 (55.1)	22 (55.0)	0.99
Hydroxyurea, *N* (%)	57 (60.6)	14 (34.1)	0.005
Transfusion dependent patients, *N* (%)	54 (48.6)	30 (65.2)	0.06
History of pulmonary hypertension, *N* (%)	1 (0.9)	0 (0.0)	0.53

Sβ-thal = Sβ-thalassemia, SCA = sickle cell anemia, *N* = number, M = male, F = female.

**Table 4:** Comparison of MRI findings between Sβ-Thal and SCA patients.

	Sβ-Thal Patients	SCA Patients	*p* Value
Left atrial area (cm^2^/m^2^)	12.6 ± 2.5	14.0 ± 3.3	0.023
Right atrial area (cm^2^/m^2^)	11.8 ± 2.0	12.2 ± 2.5	0.38
LV EDVI (mL/m^2^)	90.1 ± 19.8	93.4 ± 23.0	0.39
LV ESVI (mL/m^2^)	35.9 ± 13.6	34.6 ± 13.1	0.43
LV mass index (g/m^2^)	61.4 ± 14.8	64.6 ± 20.4	0.68
SVI (mL/m^2^)	54.3 ± 12.7	59.2 ± 14.6	0.05
Cardiac index (L/min/m^2^)	3.9 ± 1.2	3.9 ± 1.1	0.94
LV EF (%)	61.1 ± 7.2	63.1 ± 7.4	0.12
RV EDVI (mL/m^2^)	83.5 ± 19.6	86.3 ± 21.9	0.45
RV ESVI (mL/m^2^)	32.0 ± 12.2	33.3 ± 13.7	0.94
RV mass index (g/m^2^)	25.1 ± 9.1	25.5 ± 12.1	0.83
RV EF (%)	62.1 ± 8.3	62.1 ± 8.8	1.0
Global Heart T2* (ms)	36.7 ± 6.7	38.4 ± 7.2	0.15
Global Heart T2* < 20 ms, *N* (%)	2 (1.8)	0 (0.0)	0.36
At least 1 segment with T2* < 20 ms, *N* (%)	40 (36)	10 (21.7)	0.08
No MIO, *N* (%)	71 (64)	36 (78.3)	0.33
Heterogeneous MIO and no significant global heart iron, *N* (%)	38 (34.2)	10 (21.7)	
Heterogeneous MIO and significant global heart iron, *N* (%)	1 (0.9)	0 (0.0)	
Homogeneous MIO, *N* (%)	1 (0.9)	0 (0.0)	
MRI LIC (mg/g dw)	5.6 ± 6.0	7.2 ± 12.9	0.73
MRI LIC > 3 mg/g dw, *N* (%)	59 (53.2)	24 (52.2)	0.91
Myocardial fibrosis by LGE, *N* (%) (*N* = 93)	12 (17.9)	3 (11.5)	0.45

Sβ-thal = Sβ-thalassemia, SCA = sickle cell anemia, *N* = number, LV = left ventricular, EDVI = end-diastolic volume index, ESVI = end-systolic volume index, SVI = stroke volume index, EF = ejection fraction, RV = right ventricular, MIO = myocardial iron overload, LIC = liver iron concentration, LGE = late gadolinium enhancement.

## Data Availability

The data presented in this study are available on request from the corresponding author. The data are not publicly available due to privacy concerns.

## Abbreviations

Sβ-thal: Sickle beta thalassemia, SCD: sickle cell disease, RBC: red blood cell, TD: transfusion dependent, CMR: cardiac magnetic resonance, LGE: late gadolinium enhancement, RV: right ventricular, LV: left ventricular, MIO: Myocardial iron overload, SCA: sickle cell anemia, MIOT: Myocardial Iron Overload in Thalassemia, MRI: magnetic resonance imaging, EDV: biventricular end-diastolic, ESV: end-systolic volume, SV: stroke volume, EF: ejection fraction, BSA: body surface area, LIC: liver iron concentration, NTD: non-transfusion dependent, HbF: hemoglobin F, HbS: hemoglobin S, HU: hydroxyurea, Hb: hemoglobin, LDH: lactate dehydrogenase.

## References

[1] Lane P.A. (1996). Sickle Cell Disease. Pediatr. Clin. N. Am..

[2] el-Hazmi M.A., Warsy A.S., al-Swailem A.R., al-Faleh F.Z., al-Jabbar F.A. (1994). Genetic Compounds—Hb S, Thalassaemias and Enzymopathies: Spectrum of Interactions. J. Trop. Pediatr..

[3] Adekile A.D., Akbulut N., Azab A.F., Al-Sharida S., Thomas D. (2017). The Sickle β-Thalassemia Phenotype. J. Pediatr. Hematol. Oncol..

[4] Loukopoulos D. (1996). Current Status of Thalassemia and the Sickle Cell Syndromes in Greece. Semin. Hematol..

[5] Mukherjee M.B., Nadkarni A.H., Gorakshakar A.C., Ghosh K., Mohanty D., Colah R.B. (2010). Clinical, Hematologic and Molecular Variability of Sickle Cell-β Thalassemia in Western India. Indian. J. Hum. Genet..

[6] Aessopos A., Farmakis D., Trompoukis C., Tsironi M., Moyssakis I., Tsaftarides P., Karagiorga M. (2009). Cardiac Involvement in Sickle Beta-Thalassemia. Ann. Hematol..

[7] Piccin A., Murphy C., Eakins E., Rondinelli M.B., Daves M., Vecchiato C., Wolf D., Mc Mahon C., Smith O.P. (2019). Insight into the Complex Pathophysiology of Sickle Cell Anaemia and Possible Treatment. Eur. J. Haematol..

[8] Steinberg M.H. (2008). Sickle Cell Anemia, the First Molecular Disease: Overview of Molecular Etiology, Pathophysiology, and Therapeutic Approaches. ScientificWorldJournal.

[9] Serjeant G.R. (2013). The Natural History of Sickle Cell Disease. Cold Spring Harb. Perspect. Med..

[10] Meloni A., Pistoia L., Quota A., Messina G., Ricchi P., Bagnato S., Gerardi C., Lisi R., Cuccia L., Renne S. (2023). Prognostic Value of Multiparametric Cardiac Magnetic Resonance in Sickle Cell Patients. Ann. Hematol..

[11] Pepe A., Pistoia L., Gamberini M.R., Cuccia L., Lisi R., Cecinati V., Maggio A., Sorrentino F., Filosa A., Rosso R. (2022). National Networking in Rare Diseases and Reduction of Cardiac Burden in Thalassemia Major. Eur. Heart J..

[12] Varat M.A., Adolph R.J., Fowler N.O. (1972). Cardiovascular Effects of Anemia. Am. Heart J..

[13] Reddy Y.N.V., Borlaug B.A. (2016). High-Output Heart Failure in Sickle Cell Anemia. JACC Cardiovasc. Imaging.

[14] Meloni A., Pistoia L., Gamberini M.R., Cuccia L., Lisi R., Cecinati V., Ricchi P., Gerardi C., Restaino G., Righi R. (2023). Multi-Parametric Cardiac Magnetic Resonance for Prediction of Heart Failure Death in Thalassemia Major. Diagnostics.

[15] Gordeuk V.R., Bacon B.R., Brittenham G.M. (1987). Iron Overload: Causes and Consequences. Annu. Rev. Nutr..

[16] Ballas S.K. (2001). Iron Overload Is a Determinant of Morbidity and Mortality in Adult Patients with Sickle Cell Disease. Semin. Hematol..

[17] Kremastinos D.T., Farmakis D., Aessopos A., Hahalis G., Hamodraka E., Tsiapras D., Keren A. (2010). β-Thalassemia Cardiomyopathy. Circ. Heart Fail..

[18] Anderson L.J., Holden S., Davis B., Prescott E., Charrier C.C., Bunce N.H., Firmin D.N., Wonke B., Porter J., Walker J.M. (2001). Cardiovascular T2-Star (T2*) Magnetic Resonance for the Early Diagnosis of Myocardial Iron Overload. Eur. Heart J..

[19] Gladwin M.T. (2016). Cardiovascular Complications and Risk of Death in Sickle-Cell Disease. Lancet.

[20] Wood K.C., Gladwin M.T., Straub A.C. (2020). Sickle Cell Disease: At the Crossroads of Pulmonary Hypertension and Diastolic Heart Failure. Heart.

[21] Karyofyllis P., Tsiapras D., Demerouti E., Armenis I., Papadopoulou V., Voudris V. (2022). Sickle Cell Disease Related Chronic Thromboembolic Pulmonary Hypertension: Challenging Clinical Scenario. J. Thromb. Thrombolysis.

[22] Gladwin M.T., Sachdev V., Jison M.L., Shizukuda Y., Plehn J.F., Minter K., Brown B., Coles W.A., Nichols J.S., Ernst I. (2004). Pulmonary Hypertension as a Risk Factor for Death in Patients with Sickle Cell Disease. N. Engl. J. Med..

[23] Belcher J.D., Marker P.H., Weber J.P., Hebbel R.P., Vercellotti G.M. (2000). Activated Monocytes in Sickle Cell Disease: Potential Role in the Activation of Vascular Endothelium and Vaso-Occlusion. Blood.

[24] Kato G.J., Hebbel R.P., Steinberg M.H., Gladwin M.T. (2009). Vasculopathy in Sickle Cell Disease: Biology, Pathophysiology, Genetics, Translational Medicine, and New Research Directions. Am. J. Hematol..

[25] Kato G.J., Piel F.B., Reid C.D., Gaston M.H., Ohene-Frempong K., Krishnamurti L., Smith W.R., Panepinto J.A., Weatherall D.J., Costa F.F. (2018). Sickle Cell Disease. Nat. Rev. Dis. Primers.

[26] Niss O., Taylor M.D. (2017). Applications of Cardiac Magnetic Resonance Imaging in Sickle Cell Disease. Blood Cells Mol. Dis..

[27] Tsironi M., Aessopos A. (2005). The Heart in Sickle Cell Disease. Acta Cardiol..

[28] Desai A.A., Patel A.R., Ahmad H., Groth J.V., Thiruvoipati T., Turner K., Yodwut C., Czobor P., Artz N., Machado R.F. (2014). Mechanistic Insights and Characterization of Sickle Cell Disease-Associated Cardiomyopathy. Circ. Cardiovasc. Imaging.

[29] Raman S.V., Simonetti O.P., Cataland S.R., Kraut E.H. (2006). Myocardial Ischemia and Right Ventricular Dysfunction in Adult Patients with Sickle Cell Disease. Haematologica.

[30] Aquaro G.D., Camastra G., Monti L., Lombardi M., Pepe A., Castelletti S., Maestrini V., Todiere G., Masci P., di Giovine G. (2017). Reference Values of Cardiac Volumes, Dimensions, and New Functional Parameters by MR: A Multicenter, Multivendor Study. J. Magn. Reson. Imaging.

[31] Meloni A., Puliyel M., Pepe A., Berdoukas V., Coates T.D., Wood J.C. (2014). Cardiac Iron Overload in Sickle-Cell Disease. Am. J. Hematol..

[32] Junqueira F.P., Fernandes J.L., Cunha G.M., TAKubo T., MAOLima C., BPLima D., Uellendhal M., Sales S.R., ASCunha C., LR de Pessoa V. (2013). Right and Left Ventricular Function and Myocardial Scarring in Adult Patients with Sickle Cell Disease: A Comprehensive Magnetic Resonance Assessment of Hepatic and Myocardial Iron Overload. J. Cardiovasc. Magn. Reson..

[33] Wu E., Judd R.M., Vargas J.D., Klocke F.J., Bonow R.O., Kim R.J. (2001). Visualisation of Presence, Location, and Transmural Extent of Healed Q-Wave and Non-Q-Wave Myocardial Infarction. Lancet.

[34] Niss O., Fleck R., Makue F., Alsaied T., Desai P., Towbin J.A., Malik P., Taylor M.D., Quinn C.T. (2017). Association between Diffuse Myocardial Fibrosis and Diastolic Dysfunction in Sickle Cell Anemia. Blood.

[35] Moyssakis I., Tzanetea R., Tsaftaridis P., Rombos I., Papadopoulos D.P., Kalotychou V., Aessopos A. (2005). Systolic and Diastolic Function in Middle Aged Patients with Sickle Beta Thalassaemia. An Echocardiographic Study. Postgrad. Med. J..

[36] Benites B.D., Cisneiros I.S., Bastos S.O., Lino A.P.B.L., Costa F.F., Gilli S.C.O., Saad S.T.O. (2019). Echocardiografic Abnormalities in Patients with Sickle Cell/β-Thalassemia Do Not Depend on the β-Thalassemia Phenotype. Hematol. Transfus. Cell Ther..

[37] Pennell D.J., Udelson J.E., Arai A.E., Bozkurt B., Cohen A.R., Galanello R., Hoffman T.M., Kiernan M.S., Lerakis S., Piga A. (2013). Cardiovascular Function and Treatment in β-Thalassemia Major: A Consensus Statement from the American Heart Association. Circulation.

[38] Tavares A.H.J., Benites B.D., Fertrin K.Y. (2019). Myocardial Iron Overload in Sickle Cell Disease: A Rare But Potentially Fatal Complication of Transfusion. Transfus. Med. Rev..

[39] Ghoti H., Goitein O., Koren A., Levin C., Kushnir T., Rachmilewitz E., Konen E. (2010). No Evidence for Myocardial Iron Overload and Free Iron Species in Multitransfused Patients with Sickle/Beta-Thalassaemia. Eur. J. Haematol..

[40] Pepe A., Meloni A., Rossi G., Midiri M., Missere M., Valeri G., Sorrentino F., D’Ascola D.G., Spasiano A., Filosa A. (2018). Prediction of Cardiac Complications for Thalassemia Major in the Widespread Cardiac Magnetic Resonance Era: A Prospective Multicentre Study by a Multi-Parametric Approach. Eur. Heart J. Cardiovasc. Imaging.

[41] Meloni A., Ramazzotti A., Positano V., Salvatori C., Mangione M., Marcheschi P., Favilli B., De Marchi D., Prato S., Pepe A. (2009). Evaluation of a Web-Based Network for Reproducible T2* MRI Assessment of Iron Overload in Thalassemia. Int. J. Med. Inform..

[42] Mooij C.F., de Wit C.J., Graham D.A., Powell A.J., Geva T. (2008). Reproducibility of MRI Measurements of Right Ventricular Size and Function in Patients with Normal and Dilated Ventricles. J. Magn. Reson. Imaging.

[43] Marsella M., Borgna-Pignatti C., Meloni A., Caldarelli V., Dell’Amico M.C., Spasiano A., Pitrolo L., Cracolici E., Valeri G., Positano V. (2011). Cardiac Iron and Cardiac Disease in Males and Females with Transfusion-Dependent Thalassemia Major: A T2* Magnetic Resonance Imaging Study. Haematologica.

[44] Wang Y., Moss J., Thisted R. (1992). Predictors of Body Surface Area. J. Clin. Anesth..

[45] Positano V., Meloni A., Santarelli M.F., Gerardi C., Bitti P.P., Cirotto C., De Marchi D., Salvatori C., Landini L., Pepe A. (2015). Fast Generation of T2* Maps in the Entire Range of Clinical Interest: Application to Thalassemia Major Patients. Comput. Biol. Med..

[46] Meloni A., Maggio A., Positano V., Leto F., Angelini A., Putti M.C., Maresi E., Pucci A., Basso C., Marra M.P. (2020). CMR for Myocardial Iron Overload Quantification: Calibration Curve from the MIOT Network. Eur. Radiol..

[47] Meloni A., Positano V., Pepe A., Rossi G., Dell’Amico M., Salvatori C., Keilberg P., Filosa A., Sallustio G., Midiri M. (2010). Preferential Patterns of Myocardial Iron Overload by Multislice Multiecho T*2 CMR in Thalassemia Major Patients. Magn. Reson. Med..

[48] Meloni A., Luciani A., Positano V., De Marchi D., Valeri G., Restaino G., Cracolici E., Caruso V., Dell’amico M.C., Favilli B. (2011). Single Region of Interest versus Multislice T2* MRI Approach for the Quantification of Hepatic Iron Overload. J. Magn. Reson. Imaging.

[49] Positano V., Pepe A., Santarelli M.F., Scattini B., De Marchi D., Ramazzotti A., Forni G., Borgna-Pignatti C., Lai M.E., Midiri M. (2007). Standardized T2* Map of Normal Human Heart In Vivo to Correct T2* Segmental Artefacts. NMR Biomed..

[50] Cerqueira M.D., Weissman N.J., Dilsizian V., Jacobs A.K., Kaul S., Laskey W.K., Pennell D.J., Rumberger J.A., Ryan T., Verani M.S. (2002). Standardized Myocardial Segmentation and Nomenclature for Tomographic Imaging of the Heart. A Statement for Healthcare Professionals from the Cardiac Imaging Committee of the Council on Clinical Cardiology of the American Heart Association. Circulation.

[51] Wood J.C., Enriquez C., Ghugre N., Tyzka J.M., Carson S., Nelson M.D., Coates T.D. (2005). MRI R2 and R2* Mapping Accurately Estimates Hepatic Iron Concentration in Transfusion-Dependent Thalassemia and Sickle Cell Disease Patients. Blood.

[52] Pepe A., Positano V., Capra M., Maggio A., Pinto C.L., Spasiano A., Forni G., Derchi G., Favilli B., Rossi G. (2009). Myocardial Scarring by Delayed Enhancement Cardiovascular Magnetic Resonance in Thalassaemia Major. Heart.

[53] Angelucci E., Brittenham G.M., McLaren C.E., Ripalti M., Baronciani D., Giardini C., Galimberti M., Polchi P., Lucarelli G. (2000). Hepatic Iron Concentration and Total Body Iron Stores in Thalassemia Major. N. Engl. J. Med..

[54] Meloni A., Righi R., Missere M., Renne S., Schicchi N., Gamberini M.R., Cuccia L., Lisi R., Spasiano A., Roberti M.G. (2021). Biventricular Reference Values by Body Surface Area, Age, and Gender in a Large Cohort of Well-Treated Thalassemia Major Patients without Heart Damage Using a Multiparametric CMR Approach. J. Magn. Reson. Imaging.

[55] Dai D.-F., Chen T., Johnson S.C., Szeto H., Rabinovitch P.S. (2012). Cardiac Aging: From Molecular Mechanisms to Significance in Human Health and Disease. Antioxid. Redox Signal..

[56] Damy T., Bodez D., Habibi A., Guellich A., Rappeneau S., Inamo J., Guendouz S., Gellen-Dautremer J., Pissard S., Loric S. (2016). Haematological Determinants of Cardiac Involvement in Adults with Sickle Cell Disease. Eur. Heart J..

[57] Badawy S.M., Liem R.I., Rigsby C.K., Labotka R.J., DeFreitas R.A., Thompson A.A. (2016). Assessing Cardiac and Liver Iron Overload in Chronically Transfused Patients with Sickle Cell Disease. Br. J. Haematol..

[58] Meloni A., Martini N., Positano V., De Luca A., Pistoia L., Sbragi S., Spasiano A., Casini T., Bitti P.P., Allò M. (2021). Myocardial Iron Overload by Cardiovascular Magnetic Resonance Native Segmental T1 Mapping: A Sensitive Approach That Correlates with Cardiac Complications. J. Cardiovasc. Magn. Reson..

[59] Meloni A., Pistoia L., Positano V., Martini N., Borrello R.L., Sbragi S., Spasiano A., Casini T., Bitti P.P., Putti M.C. (2023). Myocardial Tissue Characterization by Segmental T2 Mapping in Thalassaemia Major: Detecting Inflammation beyond Iron. Eur. Heart J. Cardiovasc. Imaging.

[60] Meloni A., Pistoia L., Positano V., De Luca A., Martini N., Spasiano A., Fotzi I., Bitti P.P., Visceglie D., Alberini G. (2023). Increased Myocardial Extracellular Volume Is Associated with Myocardial Iron Overload and Heart Failure in Thalassemia Major. Eur. Radiol..

